# Should minimally invasive surgery be the standard approach for early-stage thymoma? A 20-year experience

**DOI:** 10.1016/j.xjtc.2025.06.007

**Published:** 2025-06-25

**Authors:** Oliver J. Harrison, Kay See Tan, Joseph Dycoco, Katherine Gray, Smita Sihag, Daniela Molena, Matthew Bott, Gaetano Rocco, James Isbell, Prasad Adusumilli, David Jones, James Huang, Valerie Rusch, Manjit Bains, Bernard Park

**Affiliations:** a Department of Thoracic Surgery, Memorial Sloan Kettering Cancer Center, New York, NY; b Department of Epidemiology and Biostatistics, Memorial Sloan Kettering Cancer Center, New York, NY

**Keywords:** thymoma, robot-assisted surgery, minimally invasive surgery, open surgery, recurrence, surgical outcomes

## Abstract

**Background:**

Minimally invasive surgery (MIS) has become the prevailing technique for resecting early-stage thymoma; however, studies evaluating the oncologic impact of this evolution are lacking. This study aimed to characterize the transition from open surgery to MIS and to evaluate outcomes.

**Methods:**

This was a retrospective, single-institution review of resections performed for pathologic TNM stage I-II thymomas between January 2000 and September 2023. The relationships between factors and recurrence were quantified using competing risk regression before and after applying overlap propensity score weights.

**Results:**

A total of 296 patients were identified, including 118 who underwent open surgery and 178 who underwent MIS (25 with video-assisted thoracic surgery and 153 with robotic-assisted thoracic surgery). Open surgery composed the vast majority of cases (92.3%) performed between 2000 and 2009, compared to 26.7% between 2010 and 2023 (*P* < .0001). Median blood loss (125 mL vs 25 mL; *P* < .0001), tumor dimension (6.5 cm vs 4.1 cm; *P* < .0001), rate of major complications (35% vs 16%; *P* = .003), and length of stay (LOS; 4 days vs 2 days; *P* < .0001) were significantly greater in the open surgery cohort. Eighteen patients developed recurrence (open, n = 12; MIS, n = 6). Univariable analyses revealed that World Health Organization class (*P* < .001), higher TNM (*P* = .043) and Masaoka-Koga stage (*P* = .017), and R1/R2 resection (*P* < .001) were associated with a greater risk of recurrence. Overlap propensity score weighting revealed a cumulative incidence of recurrence at 5 years of 5.1% (95% confidence interval [CI], 0.97%-9.2%) for open surgery (n = 47) compared to 7.1% (95% CI, 2.0%-12.9%) for MIS (n = 47), a non-statistically significant difference.

**Conclusions:**

MIS has become the principal approach for resecting early-stage thymoma over the last 2 decades. MIS is associated with a lower complication rate and shorter LOS but may be associated with a higher risk of recurrence in certain situations. Larger studies to evaluate the appropriate role of MIS are warranted.

## Graphical Abstract

**Figure:**
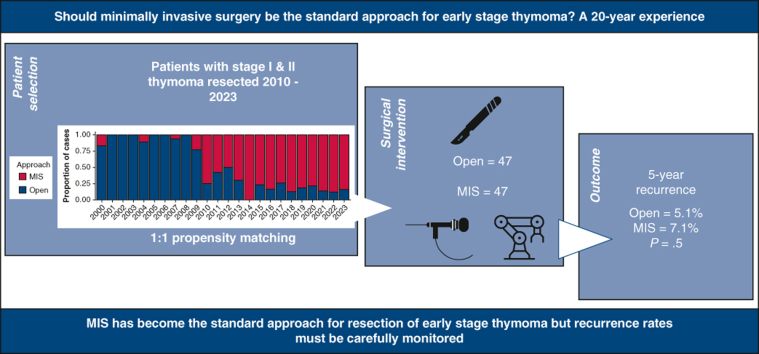

**Figure:**
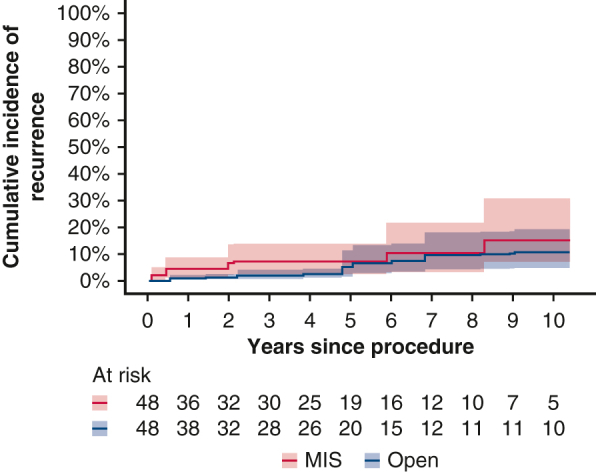
Open versus MIS thymectomy for stage I-II thymoma: propensity score–weighted recurrence rate.

**Central Message**

Minimally invasive surgery for stage I-II thymomas was associated with a marginally higher cumulative incidence of recurrence compared to open surgery. Careful consideration of the approach for certain thymomas is critical.

**Perspective**

A major shift has occurred from open surgery to minimally invasive surgery (MIS) for resection of early-stage thymoma without robust analysis of oncologic sequalae. MIS is associated with a lower complication rate and shorter length of stay but may be associated with increased recurrence in certain situations. Larger studies to evaluate the appropriate role of MIS are needed.

Thymoma is a rare malignant neoplasm of the thymic gland, and complete surgical resection represents the mainstay of curative treatment for this disease. Since the first video-assisted thoracic surgery (VATS) resection of a stage I thymoma in 1992, minimally invasive surgery (MIS), including VATS and robotic-assisted approaches, have been increasingly used for early-stage disease.^1^^,^^2^ The main advantages of these techniques include reduced operative trauma and morbidity, reduced blood loss, shorter hospital length of stay (LOS), and better cosmetic results.^2^, ^3^, ^4^ The introduction of robotic surgery in the early 2000s offered further technical advancement with 3-dimensional visualization and articulating instruments allowing for better delivery of precision surgery in small spaces, ideally suited to mediastinal diseases.^5^^,^^6^ However, given the relatively recent adoption of MIS for thymoma, many surgeons have expressed concerns regarding the risk of excessive tumor manipulation, capsular disruption, tumor seeding, and local recurrence.^7^

More recently, there has been an interest in defining the oncologic outcomes of MIS for thymoma; however, the value of this is limited, largely due to the indolent nature of the disease and long recurrence-free interval.^8^ Therefore, in this study we sought to characterize the transition from an open approach to an MIS approach and to evaluate the outcomes of this evolution over a 20-year period.

## Methods

### Patient Selection

Institutional Review Board approval was granted (approval 16-1280; approved in March 2024) to conduct this retrospective, single-institution review of a prospective surgical database of all resections for pathologic TNM (8th Edition) stage I and II thymomas performed between January 2000 and September 2023.^9^ Individual consent for publication was not required. Patients who received induction therapy were excluded. Patients were classified as either undergoing open (including median sternotomy, thoracotomy, hemi-clamshell or full clamshell) or MIS (VATS or robotic). The decision to undertake MIS was multifactorial based on the surgeon's preference and experience and on the size and extent of the tumor. The objective in all study patients was complete resection of the thymoma.

### Surgical Techniques

All techniques involved general anesthesia and intubation with a double-lumen endotracheal tube. Total thymectomy involved removing the entire thymus from the horns to the diaphragm and from phrenic to phrenic. Partial thymectomy and “excision of mediastinal mass” were defined as any subtotal thymectomy and by the operating surgeon on an individual patient basis based on achieving a complete resection. Open thymectomy was performed via a median sternotomy, thoracotomy, hemi-clamshell or full clamshell. The approach was chosen on an individual patient basis by the operating surgeon and was broadly dictated by tumor size and location. Median sternotomy, hemi-clamshell and full clamshell were performed with the patient in the supine position, and thoracotomy was performed in the lateral decubitus position. In brief, median sternotomy was made in the midline using a Hall saw to expose the thymus entirely; closure was achieved using interrupted sternal wires to approximate the sternum. Thoracotomy was performed in a posterolateral fashion in the fifth intercostal space. Hemi-clamshell was performed by partially or fully dividing the sternum and then extending the incision laterally in the fifth intercostal space, and full clamshell involved bilateral anterolateral thoracotomies in the fifth intercostal space, with division of the sternum horizontally in the midline.

MIS thymectomy was performed with the patient in a semisupine position up to 30° with a roll and the ipsilateral arm dropped below the chest to expose the axillary area and avoid instrument interference. The chest was insufflated with CO_2_ to a pressure of 8 mm Hg. For VATS thymectomy, 3 ports were used (two 5 mm and one 12 mm), along with a 30° 10-mm endoscope and an energy device based on the surgeon's preference. Specimens were extracted by extending one of the incisions and introducing an endoscopic retrieval bag. Robotic thymectomy was performed using the da Vinci system (Intuitive), with various model used throughout the study period: the Standard before 2006, S between 2006 and 2010, Si between 2010 and 2014, and Xi after 2014. Instrument choice was according to surgeon preference, and specimen extraction was performed in a similar fashion to that with VATS.

### Outcomes

The primary outcome of this study was recurrence. The time of recurrence was recorded as the date of the imaging that raised a strong suspicion, as defined by a thoracic radiologist with or without tissue confirmation in accordance with Huang and colleagues.^10^ Secondary outcomes included blood loss, resection margin status, LOS, complications, and overall survival (OS). The predominant World Health Organization (WHO) subtype identified on pathology was used to classify tumors. Cases converted from MIS to open surgery were analyzed on an intent-to-treat basis. Pulmonary comorbidities included asthma, chronic obstructive pulmonary disease, and pulmonary hypertension. Cardiac comorbidities included atrial fibrillation, hypertension, coronary artery disease, and valvular heart disease; renal comorbidities, chronic kidney disease with or without the need for dialysis. Complications were classified according to the Clavien-Dindo system.^11^ Our postoperative follow-up protocol for thymoma involves chest computed tomography scans at 6, 12, 18, and 24 months postsurgery and then annually to 5 years. Most surgeons at our institution follow patients to 10 years. The same schedule was followed over the entire 23-year study period. Thirty-nine patients (13%) did not complete the minimum 5-year follow-up for reasons other than recurrence or death.

### Statistical Analysis

Patient characteristics are summarized as median (interquartile range [IQR]) or count (percentage) and compared between the open and MIS groups using the χ^2^ and Wilcoxon rank-sum tests. OS was estimated from the time of the procedure to death due to any cause using a Kaplan-Meier approach and was compared between the 2 groups using the log-rank test. Patients were otherwise censored at the time of last follow-up. The median duration of follow-up was estimated using the reverse Kaplan-Meier approach. The incidence of recurrence between the 2 groups were summarized using cumulative incidence curves. Competing risk regression was used to quantify the relationships between factors and recurrence, with death without recurrence as a competing risk event. Propensity score weighting using overlap weights was performed to account for potential selection bias between the 2 types of procedures.^12^ The propensity scores were calculated using a logistic regression model for the event of undergoing MIS, including such factors as age, sex, WHO class, maximum tumor dimension, resection margin, Masaoka-Koga stage, T stage, pulmonary comorbidity, cardiac comorbidity, type 2 diabetes mellitus, and renal comorbidity. The overlap propensity score weighting method was then applied, in which each patient's weight was the probability of being assigned to the opposite procedure. The overlap propensity score weight method has been demonstrated to outperform conventional propensity score weighting approaches, such as inverse probability of weighting in the presence of extreme propensity scores.^12^ Covariate balance was assessed using absolute standardized mean difference before and after application of overlap weights, with a value ≤0.1 considered well-balanced between the 2 groups. The cumulative incidence of recurrence was estimated for each procedure in the overlap propensity score–weighted cohort. All statistical tests were 2-sided, and *P* < .05 was considered statistically significant. Statistical analyses were conducted with Stata version 15 (StataCorp) and R version 4.4.0 (R Core Team).

## Results

**Figure 1:**
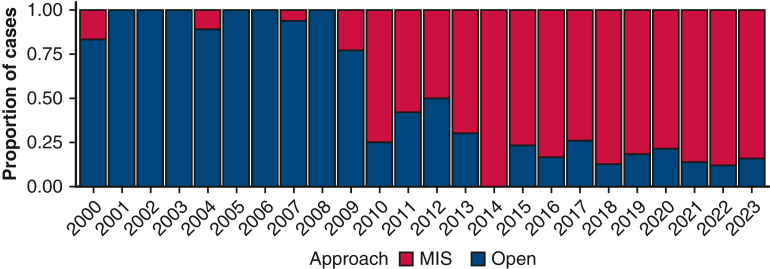
Proportion of cases performed with open surgery and with minimally invasive surgery (*MIS*) during the study period.

Patient characteristics are summarized in Table 1. The 296 study patients included 118 in the open surgery group and 178 in the MIS group (25 VATS, 153 robotic) (Table E1). Eight patients (4.5%) were converted from MIS to open surgery; reasons for conversion included inadequate exposure in 5 patients and large tumor size, obliterated pleural space, and hemorrhage in 1 patient each. Open surgery accounted for 92.3% of cases between 2000 and 2009, compared to only 24% between 2010 and 2019 (*P* < .0001; Figure 1). In the full cohort, blood loss (median, 125 mL vs 25 mL; *P* < .0001), rate of major complications (35% vs 16%; *P* = .003), and LOS (median, 4 days vs 2 days; *P* < .0001) were significantly greater in the open group. One death occurred within 30 days of surgery in the open group. The median duration of follow-up was 9.59 years (IQR, 3.49-12.80 years) in the open group and 3.04 years (IQR, 0.07-5.81 years) in the MIS group.

**Table 1:** Summary of patient and operative characteristics

Characteristic	Open group (N = 118; 40%)	MIS group (N = 178; 60%)	*P* value
Patient characteristics			
Age, y, median (IQR)	63.0 (51.0-71.0)	64.0 (53.0-70.0)	>.9
Sex, n (%)			>.9
Male	55 (47)	83 (47)	
Female	63 (53)	95 (53)	
Pulmonary comorbidity, n (%)			.4
No	106 (90)	153 (86)	
Yes	12 (10)	25 (14)	
Cardiac comorbidity, n (%)			.2
No	71 (60)	93 (52)	
Yes	47 (40)	85 (48)	
Type 2 diabetes mellitus, n (%)			.7
No	106 (90)	156 (88)	
Yes	12 (10)	22 (12)	
Renal comorbidity, n (%)			.044
No	118 (100)	171 (96)	
Yes	0 (0)	7 (3.9)	
Year of surgery, n (%)			<.001
2000-2004	26 (22)	2 (1.1)	
2005-2009	46 (39)	4 (2.2)	
2010-2014	18 (15)	42 (24)	
2015-2019	17 (14)	69 (39)	
2020-2023	11 (9.3)	61 (34)	
Operative factors			
Operative time, min, median (IQR) (N = 289)	126.0 (98.0-165.0)	139 (107.0-165.0)	.068
Procedure, n (%)			.8
Total thymectomy	105 (89)	162 (91)	
Partial thymectomy	8 (6.8)	10 (5.6)	
Excision of mediastinal mass	5 (4.2)	6 (3.4)	
Blood loss, mL, median (IQR) (N = 287)	150.0 (100.0-200.0)	25.0 (20.0-50.0)	<.001
Outcomes			
LOS, d, median (IQR) (N = 289)	4.0 (3.0-6.0)	2.0 (1.0-2.0)	<.001
Postoperative complications, n (%)			<.001
Yes	41 (35)	29 (16)	
No	77 (65)	149 (84)	
Highest-grade complication (Clavien-Dindo), n (%)			<.001
0	77 (65)	149 (84)	
1	3 (2.5)	8 (4.5)	
2	24 (20)	16 (9.0)	
3	13 (11)	4 (2.2)	
4	0 (0)	1 (0.6)	
5	1 (0.8)	0 (0)	
Major complication (grade ≥3), n (%)			.003
No	104 (88)	173 (97)	
Yes	14 (12)	5 (2.8)	
Maximum tumor dimension, cm, median (IQR)	6.50 (3.93-8.95)	4.10 (3.00-5.50)	<.001
WHO class, n (%)			.2
AB	52 (44)	80 (45)	
B1	23 (19)	22 (12)	
B2	21 (18)	33 (19)	
B3	4 (3.4)	3 (1.7)	
Mixed B	1 (0.8)	8 (4.5)	
A	17 (14)	32 (18)	
Masaoka-Koga stage, n (%)			.006
I	51 (43)	105 (59)	
IIa	17 (14)	28 (16)	
IIb	43 (36)	43 (24)	
III	7 (5.9)	2 (1.1)	
TNM stage, n (%)			.032
I	111 (94)	176 (99)	
II	7 (5.9)	2 (1.1)	
Resection margin status, n (%)			.2
R0	112 (95)	174 (98)	
R1/R2	6 (5.1)	4 (2.2)	
Postoperative radiotherapy, n (%)			.003
No	104 (88)	173 (97)	
Yes	13 (11)	5 (2.8)	
Unknown	1 (0.8)	0 (0)	

*MIS*, Minimally invasive surgery; *IQR*, interquartile range; *LOS*, length of stay; *WHO*, World Health Organization.

### Survival and Recurrence

**Figure 2:**
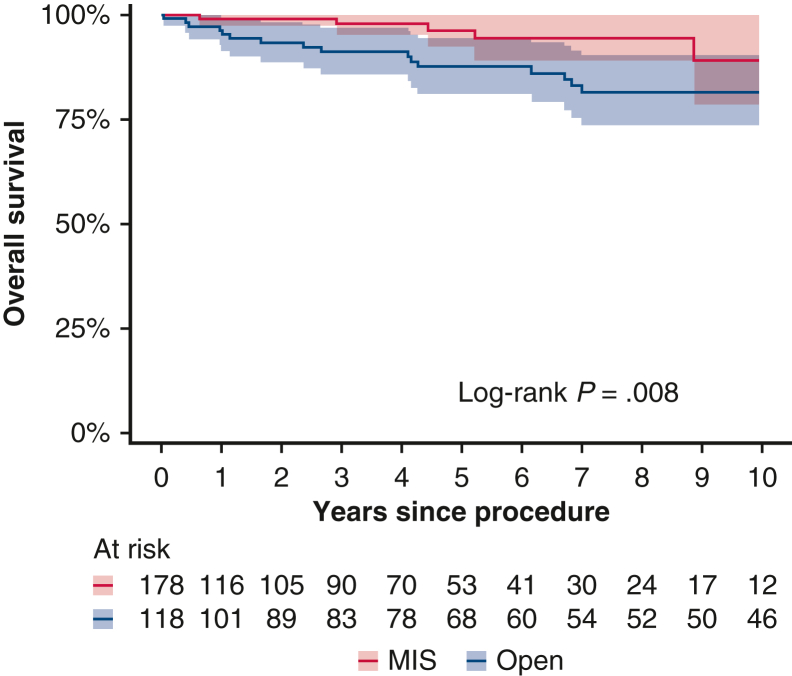
Kaplan-Meier estimates of overall survival between the minimally invasive surgery (*MIS*) and open surgery groups. The shaded bands indicate 95% confidence limits.

**Figure 3:**
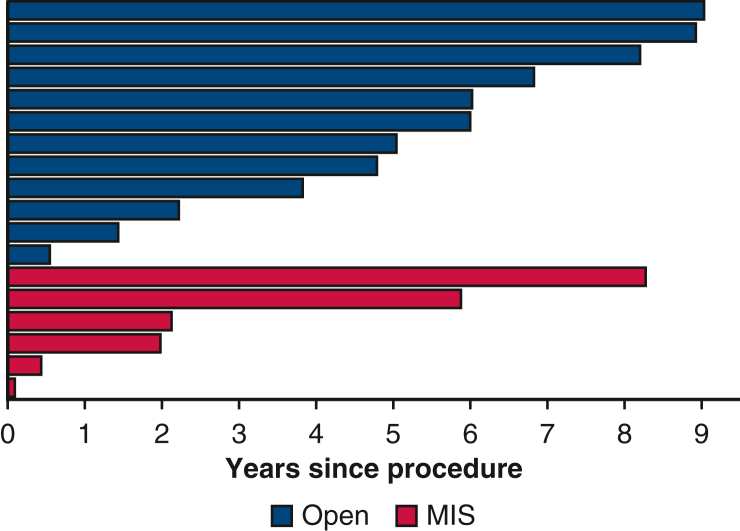
Swimmer plot for the 18 patients who experienced recurrence (open surgery group, n = 12; minimally invasive surgery [*MIS*] group, n = 6). Bar length indicates years from procedure to recurrence.

There were a total of 18 recurrences, including 12 in the open group and 6 in the MIS group. Thirty-one patients died, 28 without disease recurrence. In the full cohort, 3-year OS was 91% (95% confidence interval [CI], 86%-97%) for the open group and 98% (95% CI, 95%-100%) for the MIS group, and 5-year OS for the 2 groups was 88% (95% CI, 81%-94%) and 96% (95% CI, 93%-100%), respectively (Figure 2). Among the patients with recurrence, the median time from the procedure to recurrence was 5.5 years (IQR, 3.4-7.2 years) in the open group and 2.1 years (IQR, 0.8-4.9 years) in the MIS group (Figure 3). Six cases (33%) occurred locally in the mediastinum, and 12 cases (66%) occurred regionally in the pleural space and lung.

Univariable analysis of the full cohort demonstrated that MIS was not significantly associated with a greater risk of recurrence compared to open surgery (hazard ratio [HR], 0.68; 95% CI, 0.26-1.78; *P* = .4). WHO class (*P* < .001), higher TNM (*P* = .043) and Masaoka-Koga stage (*P* = .017), R1/R2 resection (*P* < .001), and need for postoperative radiotherapy (*P* < .001) were associated with a greater risk of recurrence (Table 2). Year of surgery was not significantly associated with recurrence (*P* = .2). Multivariable modeling was not attempted owing to a limited number of events and power.

**Table 2:** Univariable competing risk regression models for recurrence in the full cohort

Variable	Patients, n	Events, n	HR	95% CI	*P* value
Surgical approach					.4
Open	118	12	—	—	
MIS	178	6	0.68	0.26-1.78	
Age	296	18	0.99	0.95-1.02	.5
Sex					.7
Male	138	9	—	—	
Female	158	9	0.83	0.33-2.09	
Pulmonary comorbidity					<.001
No	259	18	—	—	
Yes	37	0	0.00	0.00-0.00	
Cardiac comorbidity					.7
No	164	10	—	—	
Yes	132	8	1.19	0.47-2.99	
Type 2 diabetes mellitus					.5
No	262	16	—	—	
Yes	34	2	1.72	0.39-7.59	
Renal comorbidity					<.001
No	289	18	—	—	
Yes	7	0	0.00	0.00-0.00	
Maximum tumor dimension (cm)	296	18	1.07	0.97-1.18	.2
WHO class					<.001
AB	132	3	—	—	
B1	45	5	3.55	0.86-14.7	
B2	54	6	4.40	1.07-18.0	
B3	7	2	18.0	3.36-96.6	
Mixed B	9	0	0.00	0.00-0.00	
A	49	2	1.35	0.23-7.94	
Masaoka-Koga stage					.017
I	156	3	—	—	
IIa	45	1	0.83	0.08-8.36	
IIb	86	11	4.74	1.34-16.8	
III	9	3	11.1	1.73-71.2	
TNM stage					.043
I	287	15	—	—	
II	9	3	4.79	1.05-21.9	
Resection margin status					<.001
R0	286	13	—	—	
R1/R2	10	5	9.56	3.35-27.3	
Postoperative radiotherapy					<.001
No	277	12	—	—	
Yes	18	6	6.07	2.08-17.7	
Unknown	1	0	NA	NA	
Year of surgery	296	18	0.94	0.86-1.03	.2

*HR*, Hazard ratio; *CI*, confidence interval; *MIS*, minimally invasive surgery; *WHO*, World Health Organization; *NA*, not applicable.

### Overlap Propensity Score Weighting

**Figure 4:**
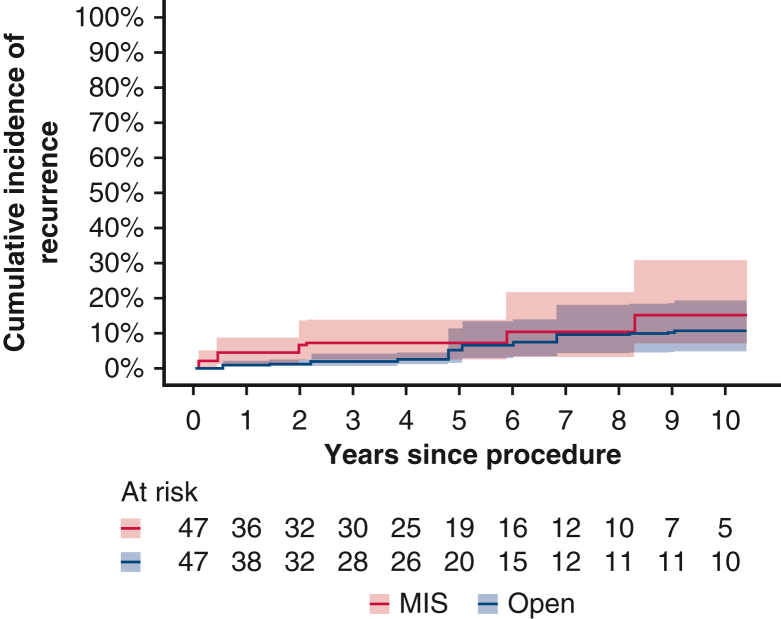
Cumulative incidence of recurrence (after overlap propensity score weighting) for the open surgery and minimally invasive surgery (*MIS*) groups. The shaded bands indicate 95% confidence limits.

The propensity score weighting procedure resulted in an effective sample size of 47 in each group, and covariate balance between the 2 groups was achieved across all variables (Table E2). In the postweighted cohort, the incidence of recurrence was higher in the MIS group; the cumulative incidence of recurrence for open surgery versus MIS was 1.8% (95% CI, 0.15%-4.1%) for open surgery and 7.1% (95% CI, 2%-12.9%) for MIS at 3 years and 5.1% (95% CI, 0.97%-9.2%) and 7.1% (95% CI, 2%-12.9%), respectively, at 5 years (Figure 4). The difference did not reach statistical significance, however.

## Discussion

Surgical resection is the gold standard treatment for thymoma when complete removal without breach of the tumor capsule can be achieved. Historically, median sternotomy was favored for thymoma of all stages and afforded unrivaled access to the thymus^13^; however, over the last decade there has been a distinct shift toward MIS techniques. A major finding of this study is the dramatic transition from open to MIS approaches for resection of early-stage thymoma that occurred between 2009 and 2010, which is consistent with data from several other high-volume centers.^2^^,^^3^ Incidentally, in the United States and Europe as a whole, this transition appears to have occurred later, toward the middle of the 2010s.^4^^,^^14^ Despite initial concerns for compromise in clinical outcomes, evidence appears to show no difference in R0 resection rate between open and MIS approaches and to indicate associations with reduced blood loss, pain, and LOS and a faster return to baseline function in patients undergoing MIS.^2^, ^3^, ^4^^,^^14^, ^15^, ^16^, ^17^, ^18^ Our data support these observations, demonstrating significantly reduced blood loss (approximately 100 mL per case), a 19% reduction in major complications, and a 2-day shorter LOS in the MIS group. Interestingly, our conversion rate for MIS was only 4.5%, which is considerably lower than the national figure of approximately 11%.^4^ Of note, in the national cohort, 11% of patients had Masaoka-Koga stage III, compared to 1% in the present study. A reduced complication rate with MIS appears to be a consistent finding in the literature, and our complication rate of 16% is comparable to the rate of 20.8% reported in a recent retrospective review.^19^

At our institution, the volume of robotic procedures greatly outnumbered VATS from an early stage in the open to MIS transition. The robotic approach is favored because our institution was an early adopter of the technology in late 2002, shortly before the first documented robotic thymectomy.^20^ Owing to the perceived technical advantages of robotic thymectomy, VATS thymectomy is now performed only rarely at our institution. A robotic approach favors surgery in confined spaces such as the mediastinum given the wristed instruments, excellent 3-dimensional visualization, and enhanced ergonomics.^21^ Although evidence is conflicting, there may be some benefits over VATS, including reduced operative time, blood loss, drainage volume, duration of chest intubation, rate of complications, and LOS (of approximately 1 day), with no difference in conversion rate.^4^^,^^22^, ^23^, ^24^, ^25^ However, the higher costs associated with robotic surgery may present a barrier to some institutions.^26^

We demonstrated a 5-year OS of 88% to 96% in our cohort, which is comparable to previously reported 5-year survival rates of 85% to 93%.^27^ Another group found no significant difference in 5-year OS between open surgery and MIS groups (88.5% [95% CI, 78.0%-94.2%] vs 90.6% [95% CI, 77.3%-96.3%]; *P* = .73, log-rank test).^4^ The reported 5-year all-cause mortality for robotic thymectomy was 90.4% in one study^19^ and 97.8% in another study.^28^

**Figure 5:**
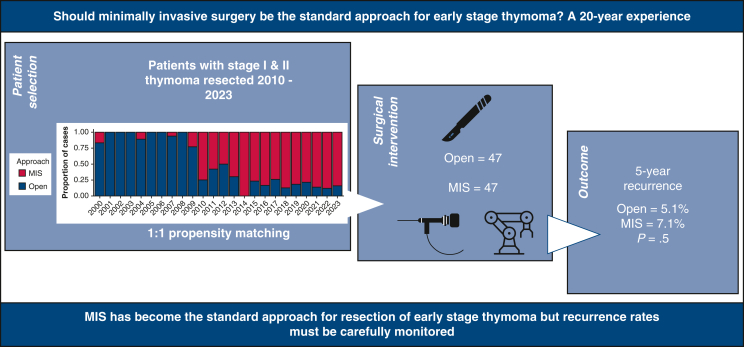
Graphical abstract. The study demonstrates a major shift over the last 2 decades from open surgery to minimally invasive surgery (*MIS*) for resecting early-stage thymoma. Our matched cohort analysis analysis suggests a 2% higher cumulative incidence of recurrence in the MIS group compared to the open group at 5 years that was not statistically significant.

Recurrence is a better measure of outcome than survival in thymoma. given the relatively indolent nature of the disease. However, studying recurrence in this type of neoplastic process is notoriously challenging, as reflected in the paucity of literature addressing the issue in early-stage thymoma after MIS resection. Reported 10-year recurrence rates in resected stage I thymoma are <5%, rising to roughly 10% in stage II.^27^ Although not statistically significant, a key finding of this study is the 5% higher cumulative incidence of recurrence at 3 years and the 2% higher incidence at 5 years for MIS compared to open surgery in the matched cohort analysis (Figure 5). This observation should be interpreted with caution but certainly merits further attention and investigation in a larger multicenter study. A higher recurrence rate has been suggested with robotic surgery compared to open surgery in the matched cohort analysis in a study of gynecologic surgery, although this represents a very different situation than robotic thymoma resection.^29^ Increased tumor manipulation was suggested as a possible cause.

In thymomas, where seeding through capsule violation is a recognized phenomenon, transition from open surgery to MIS, along with a possible learning curve effect, may be hypothesized to increased recurrence. This has not been borne out in the literature to date, however, with all available studies showing no difference in recurrence rate between open surgery and MIS.^3^^,^^14^^,^^30^, ^31^, ^32^ Adequate matching in such studies is important to minimize the risk of type 2 error, particularly given the likely far longer follow-up for open cases compared to MIS cases, as seen in the present study. When recurrence occurs, it does so almost exclusively in the chest, with approximately one-half of cases occurring locally in the mediastinum and the other half occurring regionally in the lung or pleural space.^27^ This was broadly reflected in our present findings.

Complete removal with negative margins is perhaps the most important factor when resecting any thymoma and was well described by Toker and colleagues^7^ at the beginning of the MIS transition. Fundamentally, MIS should not violate the principles of sound thymoma surgery—ensuring a sufficiently large access incision to prevent specimen disruption, retrieving the specimen in a bag, examining the specimen to ensure completeness of resection, and having a low threshold for conversion to open surgery if oncologic principles are compromised (eg, capsule disruption, incomplete resection). For the most part, these principles appear to hold true for MIS in the literature to date. Even in the setting of stage II and III disease, comparable survival to stage I is seen when an R0 resection is achieved.^33^ Concurrently, our study demonstrates that an R1/R2 resection, higher TNM and Masaoka-Koga stage, and tumor subtype B2 and B3 were significantly associated with recurrence in univariable analysis which is consistent with previous studies.^34^^,^^35^ An analysis of the International Thymic Malignancy Interest Group worldwide database identified thymoma WHO class as a prognostic factor in stage I to II (1473 cases with R0 resection status), as was achieving an R0 resection.^36^ R1 resection conferred a 1.64-fold increased risk of recurrence. Our data suggest the need for caution when resecting larger tumors and tumors with type B2 or B3 histology, although the nature of early-stage thymoma rarely necessitates preoperative biopsy. Instead, the principles of Toker and colleagues^7^ should be respected, with every tumor treated as potentially having a B2 or B3 histology.

Other groups have suggested that tumors >6 cm should be considered for an open approach to minimize the risk of inadvertent capsule rupture and increased risk of recurrence^3^^,^^37^; however, tumors as large at 15.5 cm have been excised with MIS. Similarly, a recent retrospective review by Huang and colleagues^38^ demonstrated no association between tumor size and prognosis (including relapse-free survival and OS) when using thresholds of both 5-cm and 8-cm for robotic resection of large thymomas. Tumor size does not appear to be as important as other factors in achieving complete resection in MIS.^2^ Indeed, in the present study, univariable regression analysis did not demonstrate a correlation between recurrence and tumor size. Of note, 61 patients (34%) of the MIS group had a maximum tumor dimension ≥5 cm, and 3 patients had a maximum dimension >10 cm. None of these 3 patients had recurrence.

Our data suggest that both TNM and Masaoka-Koga stage are associated with recurrence in early stage thymoma. Interestingly, Masaoka-Koga stage may be better suited to early stage thymoma given the wider distribution of patients across the Masaoka-Koga stages I, IIa and IIb which by TNM would simply be stage I.

The results of this study must be interpreted with caution given several limitations, including the inherent biases associated with its retrospective design and reflection of only a single-center experience. A rigorous attempt was made to reduce bias and account for confounding variables by univariable modeling and propensity-score weighting. Even though our institution is a high-volume center with data spanning more than 2 decades, our case numbers for recurrence were low, which limited our ability to characterize this group of patients in more detail. The duration of follow-up for our cohort was relatively long compared to previous studies; however, cases toward the end of the study period had shorter follow-up and were more likely to be MIS, which might have introduced bias. Another potential source of bias may be tumor location in the superior or inferior mediastinum, which we did not account for but may be associated with risk of recurrence.^39^

Limitations notwithstanding, to the best of our knowledge our study represents the largest reported single-center experience for resection of early-stage thymoma with both open and MIS techniques. We believe that our present data suggest that recurrence rate may be an important factor to consider in future larger multicenter studies as the world continues its transition to MIS for the resection of early-stage thymoma.

In conclusion, the last 2 decades have seen a major shift from open surgery to MIS for the resection of early-stage thymoma. MIS is associated with less blood loss, a lower complication rate, and shorter LOS but may be associated with a higher risk of recurrence in certain situations, particularly in the presence of B2 and B3 histology and higher Masaoka-Koga stage. Larger multicenter collaborations may help evaluate the appropriate role of MIS.

## Conflict of Interest Statement

Dr Park serves as a consultant for CEEVRA and receives speaking honoraria from Medtronic, Intuitive Surgical, and BD. Dr Jones is a member of the advisory council for AstraZeneca and the advisory committee for More Health, has been a speaker for DAVA Oncology, and receives research grant support from Merck. All other authors reported no conflicts of interest.

The *Journal* policy requires editors and reviewers to disclose conflicts of interest and to decline handling or reviewing manuscripts for which they may have a conflict of interest. The editors and reviewers of this article have no conflicts of interest.

## Figures and Tables

**Table E1:** Operative approaches for the open surgery and MIS groups

Operative approach	Open surgery group, n (%)	MIS group, n (%)
Median sternotomy	83 (70)	0 (0)
Thoracotomy	16 (14)	0 (0)
Hemi-clamshell	17 (14)	0 (0)
Full clamshell	2 (1.7)	0 (0)
VATS	0 (0)	23 (13)
RATS	0 (0)	148 (83)
RATS converted to open	0 (0)	5 (2.8)
VATS converted to open	0 (0)	2 (1.1)

*MIS*, Minimally invasive surgery; *VATS*, video-assisted thoracic surgery; *RATS*, robotic-assisted thoracic surgery.

**Table E2:** Patient characteristics before and after propensity score matching using inverse probability of treatment weighting

Characteristic	Before matching			After matching		
	Open group	MIS group	SMD	Open group	MIS group	SMD
Number of patients	118	178		47	47	
Age, y, median (IQR)	63.00 (51.25-71.00)	64.00 (53.00-70.00)	0.068	63.00 (53.00-72.60)	62.00 (52.00-69.00)	<0.001
Female sex, n (%)	63 (53.4)	95 (53.4)	<0.001	27 (56.8)	27 (56.8)	<0.001
Pulmonary comorbidity, n (%)	12 (10.2)	25 (14.0)	0.119	6 (13.6)	6 (13.6)	<0.001
Cardiac comorbidity, n (%)	47 (39.8)	85 (47.8)	0.160	21 (43.2)	21 (43.2)	<0.001
Type 2 diabetes mellitus, n (%)	12 (10.2)	22 (12.4)	0.069	5 (10.4)	5 (10.3)	0.003
Renal comorbidity, n (%)	0 (0.0)	7 (3.9)	0.286	0 (0.0)	0 (0.0)	<0.001
WHO class, n (%)			0.323			<0.001
AB	52 (44.1)	80 (44.9)		23 (48.3)	23 (48.3)	
B1	23 (19.5)	22 (12.4)		8 (16.3)	8 (16.3)	
B2	21 (17.8)	33 (18.5)		8 (17.3)	8 (17.3)	
B3	4 (3.4)	3 (1.7)		1 (2.5)	1 (2.5)	
Mixed B	1 (0.8)	8 (4.5)		0 (0.0)	0 (0)	
A	17 (14.4)	32 (18.0)		7 (15.0)	7 (15.0)	
Maximum tumor dimension, cm, median (IQR)	6.50 (3.92-8.95)	4.10 (3.00-5.50)	0.768	5.20 (3.50-7.50)	5.00 (3.60-6.98)	<0.001
Resection margin R1/R2, n (%)	6 (5.1)	4 (2.2)	0.151	1 (2.1)	1 (2.1)	<0.001
Masaoka-Koga stage, n (%)			0.415			<0.001
1	51 (43.2)	105 (59.0)		24 (50.2)	24 (50.2)	
2a	17 (14.4)	28 (15.7)		8 (17.2)	8 (17.2)	
2b	43 (36.4)	43 (24.2)		14 (30.1)	14 (30.1)	
3	7 (5.9)	2 (1.1)		1 (2.6)	1 (2.6)	
T stage, n (%)			0.291			<0.001
1a	106 (89.8)	172 (96.6)		44 (93.3)	44 (93.3)	
1b	5 (4.2)	4 (2.2)		2 (4.1)	2 (4.1)	
2	7 (5.9)	2 (1.1)		1 (2.6)	1 (2.6)	
TNM stage II, n (%)	7 (5.9)	2 (1.1)	0.263	1.2 (2.4)	1.2 (2.4)	<0.001
Recurrence, n (%)	12 (10.2)	6 (3.4)		3 (6.4)	4 (8.5)	

*MIS*, Minimally invasive surgery; *SMD*, standardized mean difference; *IQR*, interquartile range; *WHO*, World Health Organization.
